# Comparing supervised machine learning algorithms for the prediction of partial arterial pressure of oxygen during craniotomy

**DOI:** 10.1186/s12911-025-03148-8

**Published:** 2025-09-03

**Authors:** Andrea S. Gutmann, Maximilian M. Mandl, Clemens Rieder, Dominik J. Hoechter, Konstantin Dietz, Benjamin P. Geisler, Anne-Laure Boulesteix, Roland Tomasi, Ludwig C. Hinske

**Affiliations:** 1 Department of Anaesthesiology, LMU University Hospital, LMU Munich, Munich Germany; 2 Institute for Medical Information Processing, Biometry and Epidemiology (IBE), Faculty of Medicine, LMU Munich, Pettenkofer School of Public Health, Munich, Germany; 3 Institute for Medical Information Processing, Biometry and Epidemiology (IBE), Faculty of Medicine, LMU Munich, Munich, Germany; 4 Munich Center for Machine Learning, LMU Munich, Munich, Germany; 5 Department of Health Management and Health Economics, University of Oslo, Oslo, Norway; 6 Institute for Digital Medicine, University Hospital of Augsburg, Augsburg, Germany

**Keywords:** Machine learning, paO_2_, Craniotomy, Blood gas analysis, Arterial partial pressure of oxygen

## Abstract

**Background and Objectives:**

Brain tissue oxygenation is usually inferred from arterial partial pressure of oxygen (paO_2_), which is in turn often inferred from pulse oximetry measurements or other non-invasive proxies. Our aim was to evaluate the feasibility of continuous paO_2_ prediction in an intraoperative setting among neurosurgical patients undergoing craniotomies with modern machine learning methods.

**Methods:**

Data from routine clinical care of lung-healthy neurosurgical patients were extracted from databases of the respective clinical systems and normalized. We used recursive feature elimination to identify relevant features for the prediction of paO_2_. Six machine learning regression algorithms (gradient boosting, k-nearest neighbors, random forest, support vector, neural network, linear model with stochastic gradient descent) and a multivariable linear regression were then tuned and fitted to the selected features. A performance matrix consisting of standard deviation of absolute errors (**σ**_ae_), mean absolute percentage error (MAPE), adjusted R^2^, root mean squared error (RMSE), mean absolute error (MAE) and Spearman’s **ρ** was finally computed based on the test set, and used to compare and rank each algorithm.

**Results:**

We analyzed *N* = 4,581 patients with *n* = 17,821 observations. Between 5 and 22 features were selected from the analysis of the training dataset comprising 3,436 patients with 13,257 observations. The best algorithm, a regularized linear model with stochastic gradient descent, could predict paO_2_ values with σ_ae_ = 86.4 mmHg, MAPE = 16 %, adjusted R^2^ = 0.77, RMSE = 44 mmHg and Spearman’s ρ = 0.83. Further improvement was possible by calibrating the algorithm with the first measured paO_2_/FiO_2_ (p/F) ratio during surgery.

**Conclusion:**

PaO_2_ can be predicted by perioperative routine data in neurosurgical patients even before blood gas analysis. The prediction improves further when including the first measured p/F ratio, realizing quasi-continuous paO_2_ monitoring.

**Supplementary Information:**

The online version contains supplementary material available at 10.1186/s12911-025-03148-8.

## Introduction

It has long been known that brain tissue is exquisitely sensitive to decreased levels of blood oxygen, leading to potentially irreversible damage, including brain death within minutes [1]. Consequently, hypoxemia has been the subject of extensive research since the mid-19^th^ century, enabling a deeper understanding of its mechanisms and the development of life-saving interventions [2]. Hyperoxemia, an elevated level of oxygen in the blood, has been less extensively studied, even though it might occur very frequently in clinical practice through supplemental oxygen. In the last few years, however, it has been claimed in the literature that oxygen’s inherently reactive nature may damage lipids, proteins, nucleic acids, and thus hyperoxemia may lead to acute lung, kidney, and myocardial injury, increased mortality, pulmonary complications, and cardio- and cerebrovascular complications [2–4]. Suzuki et al. estimate that more than 80% of patients undergoing general anesthesia are exposed to amounts of supplemental oxygen exceeding levels necessary to maintain a normal blood oxygen saturation [3, 5]. Diagnosing hyperoxemia is more difficult as its initial symptoms - if any - may be vague [2, 6], and also because confirmation requires an arterial blood draw. In contrast, hypoxemia can often be diagnosed with peripheral pulse oximetry, which is non-invasive. Consequently, hyperoxemia is the subject of less extensive research. The lack of consensus regarding potential oxygen over-supplementation highlights the need for further research and guidelines in clinical practice [6–10].

Arterial blood gas analysis ABG is performed during surgeries and in the intensive care unit, allowing the indirect monitoring of gas exchange in the lungs, tissue oxygenation, and oxygen consumption. However, these measurements are only valid at the time of each arterial blood draw, which, due to their invasive nature, is infrequently performed. To overcome this limitation, several approaches have been developed to achieve a non-invasive and continuous estimation of the partial arterial pressure of oxygen (paO_2_) as a proxy for blood oxygenation [11–17]. These methods estimate blood oxygenation using factors such as the fraction of inspired oxygen (FiO_2_) and peripheral oxygen saturation (SpO_2_) [11–13, 18], the oxygen transfer slope and estimated membrane oxygen transfer [14], the alveolar gas equation (pAO_2_) [15] or venous blood gas samples [16, 17, 19, 20]. However, it is important to note that our analysis reveals that none of these methods demonstrate a particularly high level of precision, and they also exhibit other limitations, such as constraints related to the formula they utilize.

Machine learning algorithms may be able to overcome these limitations and potentially perform better when predicting outcomes based on a higher number of features, non-linear effects, and complex association patterns [21–23].

Given the brain tissue’s sensitivity to hypoxia, neurosurgical patients may be regularly administered excessive amounts of oxygen to increase the margin of safety in case of an emergency [4]. This cohort consists of lung-healthy patients who undergo frequent ABG analyses compared to other interventions, providing a larger data pool. Therefore, our study aimed to demonstrate that machine learning algorithms outperform surrogate parameters or existing equations in calculating paO_2_ values for neurosurgical patients, achieving a satisfiable range of error and good performance parameters. Additionally, we aimed to identify the most accurate machine learning algorithm for near-continuous prediction of paO_2_ values.

## Materials and methods

### Data and data preparation

The study was conducted as a single-left retrospective cohort study. Before accessing the data, our protocol (submission 19–539) received approval from the University of Munich’s institutional review board and consent was waived.

We included all patients at the University Hospital of Munich between January 1^st^ 2008 and December 31^st^ 2019 undergoing craniotomy as identified by the German surgical procedure classification [24] codes 5–01 or 5–02 being at least 18 years old. Further inclusion criteria were as follows: receiving general anesthesia with endotracheal intubation, with documented anesthesia induction, incision, closure, and termination of anesthesia times, and having at least two perioperatively paO_2_ measurements. In cases where a patient had undergone multiple surgeries, the one with most paO_2_ measurements was included for analysis. The minimum number of patients required for the study was calculated applying the formula $N=\frac{L}{f^2}+k+1$, with *k* = 23 (number of available features), $f^2=0.02$ (small effect) and *L* = 27.94 for *α* = 0.05 and *β* = 0.9 [25, 26].

Data were extracted and integrated from the Anesthesia Information Management System (NarkoData®, IMESO IT GmbH, Giessen, Germany) and the Hospital Information System (SAP/Cerner i.s.h.med, Idstein, Germany) prior to data anonymization.

Hemoglobin and pH values were included regardless of their sampling site and without transformations [27, 28]. Variables known to impact oxygenation were pre-selected. Additionally, we included the underlying physiologic model of the alveolar gas equation (pAO_2_) [15] as well as the intraoperatively measured paO_2_/FiO_2_ (p/F) ratio as an indicator of pulmonary function. Ventilation compliance, represented by static compliance, was incorporated in our analysis as well [29]. Finally, we calculated a paO_2_ value based on Gadrey et al. [30]. The formulas are stated in appendix A. Each observation was defined as a set of multiple measurements at the same time, encompassing the aforementioned features, as well as one-time measures, such as socio-demographic information. The complete set of variables is provided in appendix B. Of note, more than one observation was collected throughout each surgical procedure. Thus, multiple paO_2_ values were predicted for each patient.

A detailed description of inclusion and exclusion criteria is provided in appendix C.

Little’s test for data missing completely at random was conducted [31], yielding no significant p-values. This supports the use of complete case analysis [32]. Still, missing values in pre-operative creatinine and temperature measurements were systematic in nature. Specifically, temperature data can be absent because the urinary catheter-and with it, the temperature probe-is typically placed as one of the final pre-surgical steps. In contrast, ABG analyses are frequently performed earlier, resulting in some missing temperature readings. Similarly, pre-operative creatinine values were not consistently recorded, as these measurements are not routinely ordered for all patients. Following the application of exclusion criteria, missing values for both parameters were iteratively imputed, using median values for initialization. Imputed pre-operative creatinine values were averaged across each patient to obtain a single value.

All features (independent variables) and labels (dependent variable) were normalized before analysis by scaling them to a range between 0 and 1. The formula is stated in appendix A.

### Algorithms

Six machine learning algorithms and a multivariable linear regression (MLR), which was used as a reference model, were used for feature selection and hyperparameter tuning. The best model, based on the performance matrix, was used for further evaluation. The employed machine learning algorithms were:

- Gradient Boosting for Regression (GBR),
- Regression based on k-nearest neighbors (KNN),
- Random Forest Regressor (RFR),
- Epsilon-Support Vector Regression (SVR),
- Linear model fitted by minimizing a regularized empirical loss with stochastic gradient descent (SGD),
- Multi-layer Perceptron Regressor (MLP).

None of these algorithms handle longitudinal paired data by default. To account for this, we added subject IDs and time points of measurements. All cross-validations are performed as group cross-validation, i.e. by taking the cluster structure into account (the measurements of one patient building a “cluster”), as commonly recommended in the literature [33]. Thus, all measurements of the same patient were assigned to the same fold, in order to avoid leakage between training and test data.

### Training and test datasets

**Fig. 1:**
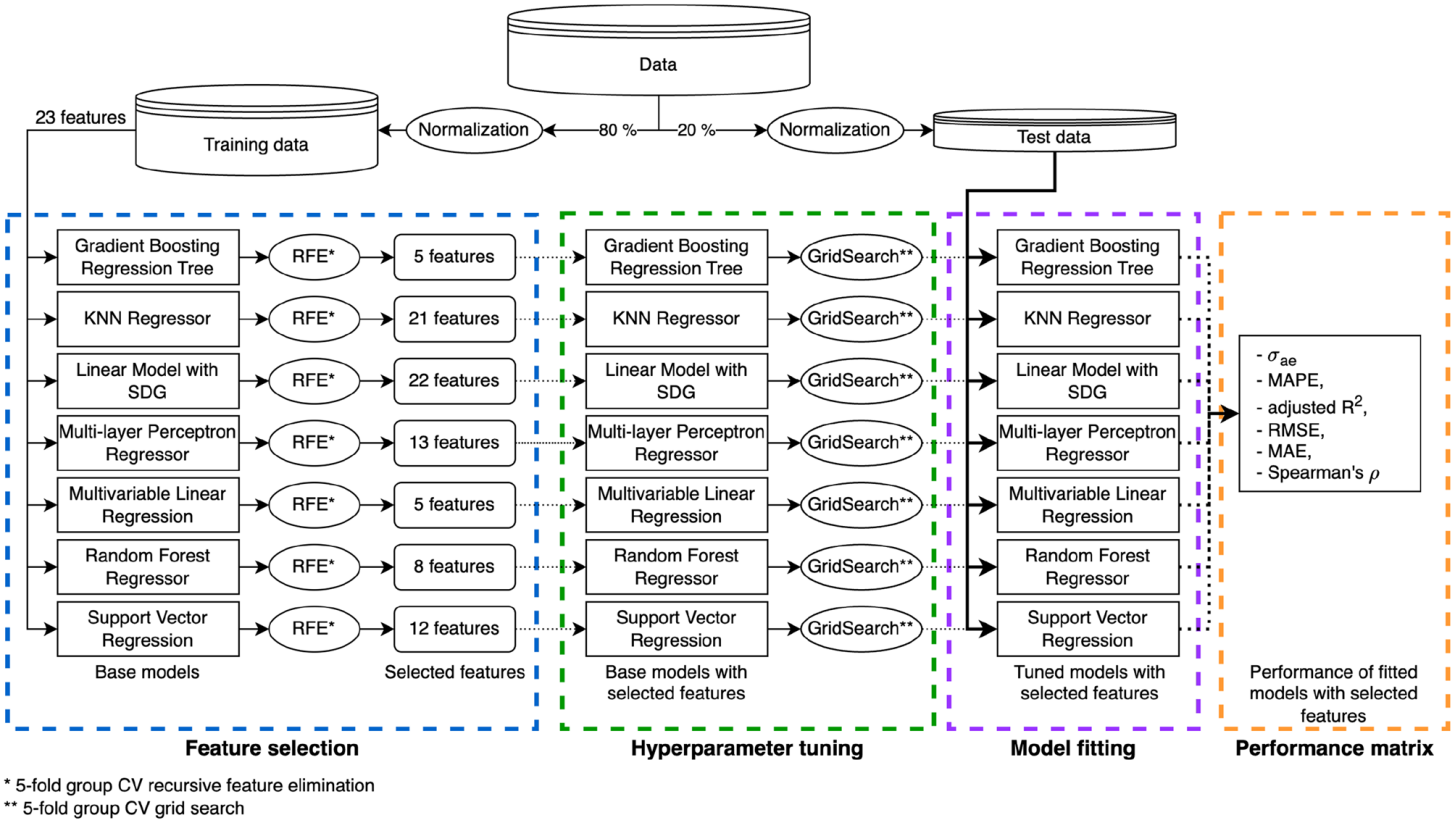
Methods flowchart. Use of data in training and testing during feature selection, hyperparameter tuning, model fitting and performance evaluation

The dataset was randomly divided by an 8:2 ratio into a training and test set, preventing an overoptimistic bias in performance evaluation. The feature selection process and hyperparameter tuning of all algorithms were conducted exclusively on the training set (Fig. 1). The test set was reserved solely to calculate the performance metrics. The same set was used for evaluating feature importance, percentage errors and binning.

### Feature selection

To optimize each algorithm effectively, a two-step feature selection process was performed individually for each model. The optimal number of features was determined via five-fold group cross-validation (CV) on the training set, with patients randomly assigned to one of the folds. Model performance was evaluated using the pooled negative standard deviation of absolute errors (σ_ae_). To maintain compatibility with the scikit-learn package, performance metrics intended to be minimized were multiplied by −1.

In the first step, the goal was to identify the optimal number of features by balancing computational efficiency (fewer features) against predictive performance (cross-validated σ_ae_). For each algorithm, we calculated the percentage improvement of the pooled σ_ae_ between successive feature counts. When this improvement exceeded 50%, the corresponding score was recorded as the point of substantial improvement. The lowest of these scores across all algorithms was then used as a global threshold to uniformly determine the optimal number of features.

In the second step, the features themselves were selected using recursive feature elimination (RFE), with each regressor serving as its own estimator. Feature importance rankings were computed on the full training set, constrained to the previously determined optimal number of features. The top-ranked features were then used for hyperparameter tuning and final model training.

### Tuning of machine-learning algorithms and model fitting

Hyperparameter tuning was performed using group cross-validation with a grid search or - in case of high computational costs - a randomized search (for MLP only) across all prespecified parameter combinations within the training set. For each algorithm and hyperparameter setting, 5-fold group CV was applied. In each fold, negative σ_ae_, negative mean absolute percentage error (MAPE), adjusted R^2^, and negative root mean squared error (RMSE) were recorded, among other parameters like fitting and scoring time or ranks of the averaged metrics. These metrics were aggregated as means and standard deviations per hyperparameter combination. A composite score was then computed as the weighted sum of pooled σ_ae_, pooled MAPE, pooled negative adjusted R^2^, and pooled RMSE with weights of 0.5, 0.3, 0.1 and 0.1, respectively. The best model was selected based on the lowest composite score.

Details about the architectures of all algorithms are reported in appendix D.

### Performance evaluation

For each base and tuned algorithm, a performance matrix consisting of six metrics was calculated on the whole test set. These metrics were (1) σ_ae_, (2) MAPE, (3) adjusted R^2^, (4) RMSE, (5) mean absolute error (MAE), and (6) Spearman’s rank correlation coefficient ρ [13, 30, 34–36].

For each algorithm, we ranked the quality measure of the tuned estimator from one for best to seven for worst based on the test set. These ranks were summed up as the overall rank for each algorithm, and the best one was determined by the lowest overall rank.

### Further evaluation

The agreement between measured and predicted paO_2_ values was evaluated using a Bland-Altman plot [37]. Feature importance was evaluated using SHapley Additive exPlanations (SHAP) values [38, 39].

We grouped all measured and predicted paO_2_ values into bins spanning 50 mmHg based on the measured paO_2_ value. Any values above 450 mmHg were grouped into a single bin, while all values below 100 mmHg were also grouped into a single bin. For each bin, the mean and standard deviation of the observed and the predicted paO_2_ values was calculated.

Outliers were defined based on a combination of the Percentage Error ($$PE=\frac{A_i-P_i*100}{A_i}$$), the interquartile range (IQR), first quartile and the third quartile (Q1 / Q3): $$outliers:=PE <$$$$[Q1-1.5*IQR; Q3+1.5*IQR]$$$$< PE$$ [40]. We investigated all corresponding observations to detect differences between highly over- or underestimated paO_2_ values.

At last, the best algorithm was retrained with the first measured p/F ratio of each patient as an additional feature to assess whether the prediction of paO_2_ values could be further improved. The same test set was used to calculate the performance measures.

### Implementation, reproducibility, and reporting

Data extraction, processing and analysis were done in Python on three different systems. Data were extracted on system 1, and statistical analyses were performed on system 2. All cross-validated recursive feature eliminations and grid searches were performed on system 3. Information about the systems and their operating systems as well as a complete list of each package version used in each system can be found in appendix F.

Extensive reporting for the prediction model development was done using the Transparent Reporting of a multivariable prediction model for Individual Prognosis Or Diagnosis checklist (appendix G). The results of the study were reported following the guideline as provided by The Strengthening the Reporting of Observational Studies in Epidemiology in appendix H.

## Results

### Patient cohort

The calculated required sample size for the study was 1,421 patients. During the study period, 6,027 intracranial surgeries (number of surgeries, N) with 25,032 observations (number of perioperative value sets comprising two ABG analyses and all corresponding ventilation and surgical parameters as well as demographic data and vital signs, n) met the inclusion criteria. 9,125 observations and 1,484 surgeries were excluded based on our other criteria, resulting in a final data set of 4,581 surgeries with a total of 17,821 observations (appendix C). 2,140 and 1,385 temperature and preoperative creatinine values were imputed before splitting into training and test sets. The training set included 3,436 surgeries with 13,257 observations and the test set 1,145 surgeries with 4,564 observations.

The patients mean age was 54 years. Among the patients, 56% were female, and the mean body mass index (BMI) was 25.1 kg/m^2^. The mean ventilation time was 353 min, the mean incision to closure time was 243 min. Before applying the exclusion criteria, patients received an average of 4.15 ABG analyses during the ventilation period. After applying the criteria, the average number of ABG analyses per patient decreased to 3.89. The mean initial p/F ratio was 461.5. The minimum measured paO_2_ value was 33 mmHg, while the mean value was 212 mmHg with a standard deviation of 91 mmHg. Twelve measurements from eleven patients showed a paO_2_ <60 mmHg. Nine of these ABG analyses were obtained around the time of intubation, when oxygen supply was established via mechanical ventilation. 171 patients had an American Society of Anesthesiologists (ASA) class of I, 1,752 had ASA class II, 2,118 had ASA class III, 496 had ASA class IV and 44 had ASA class V. The mean postoperative length-of-stay was 11.6 days. A detailed description as well as differences between the training and test set can be found in appendix I.

### Feature selection

**Fig. 2:**
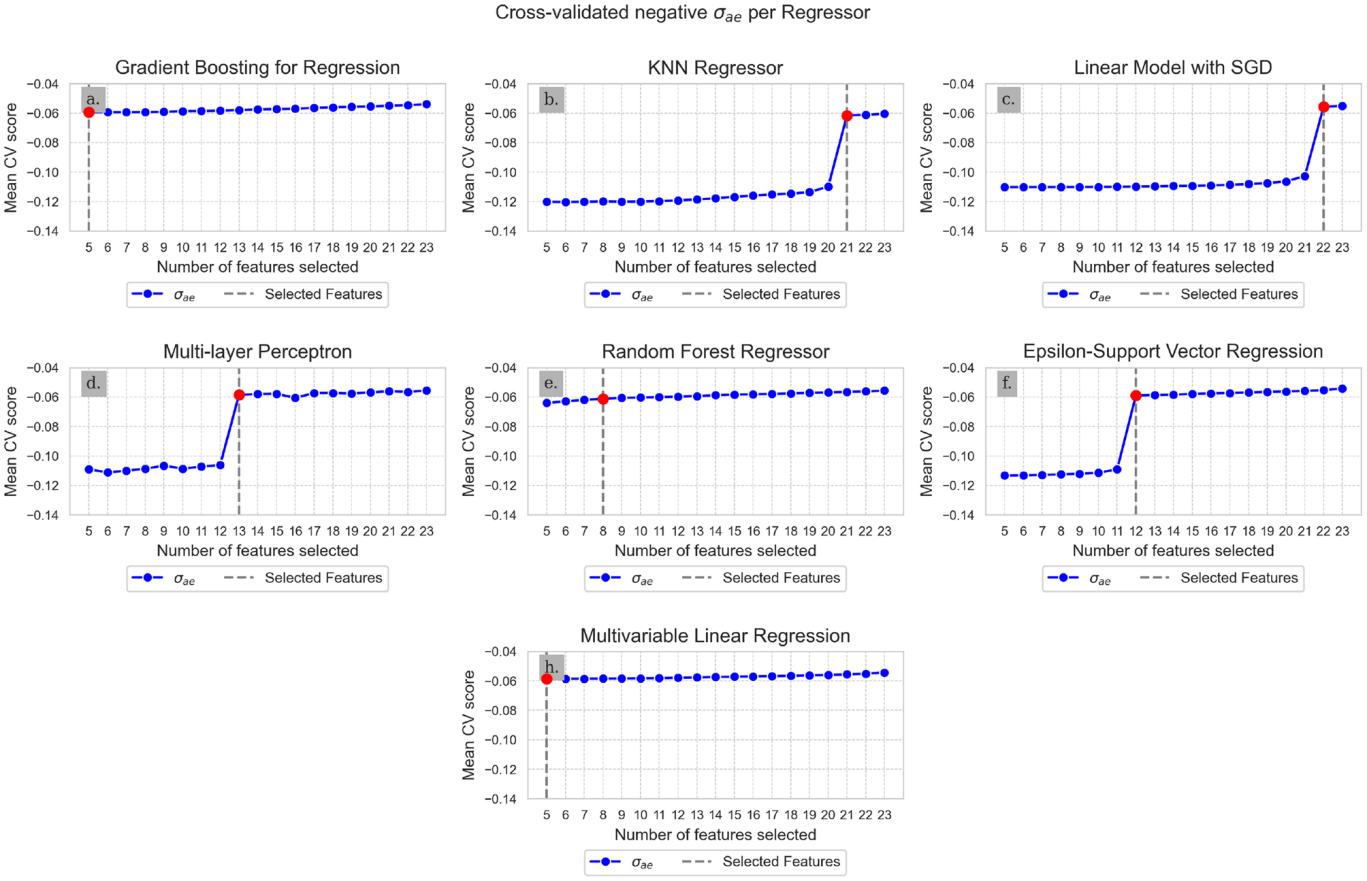
Cross-validation scores (negative σ_ae_) for n number of features. The red dot indicates the selected number of features

The feature selection started with 23 variables (see appendix B). The calculated negative σ_ae_ during cross-validated RFE for the scaled training set is shown in Fig. 2.

For every regressor, the highest negative σ_ae_ score was reached with 23 features. The appropriate number of features was selected at the first point at which the negative σ_ae_ exceeded −0.06158, which is indicated by the red vertical line on every plot in Fig. 2.

### Comparison to popular proxies and base models

**Fig. 3:**
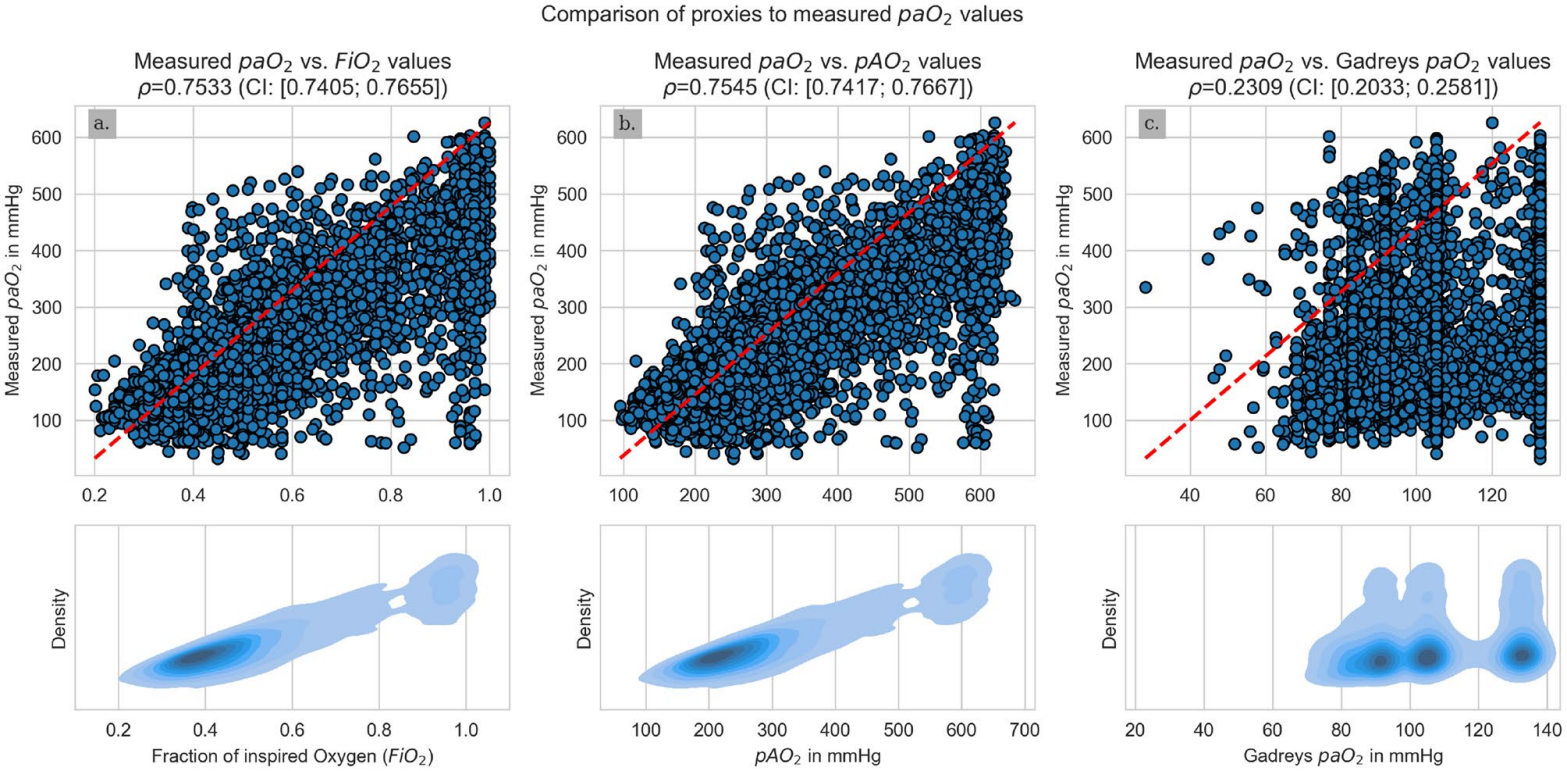
Comparison of paO_2_ measurements to popular proxies. The first row shows scatter plots of the parameter evaluated on the x-axis vs. The mean measured pAO_2_ and the second row shows the density plots corresponding to the first row. First column: FiO_2_; second column: pAO_2_; third column: Gadrey’s paO_2_

The correlation coefficients of averaged FiO_2_, pAO_2_, and Gadrey’s paO_2_ to the averaged measured paO_2_ were 0.75, 0.75 and 0.23 (Fig. 3).

All base models (BM) for the paO_2_ prediction (not-tuned regressors with default parameters) reached MAPEs between 17 and 20%, σ_ae_ between 83 and 89 mmHg (for rescaled data), RMSEs of 51 mmHg and less (for rescaled data), MAEs of less than 87 mmHg (for rescaled data), adjusted R^2^ between 0.70 and 0.76, correlation coefficients ρ between 0.74 and 0.82 with confidence intervals ranging from 0.74 to 0.83. Therefore, all BMs showed at least the same correlation coefficients between measured and predicted paO_2_ values as the FiO_2_ or pAO_2_ values alone and a better correlation than Gadrey’s paO_2_.

### Tuned algorithms

Following hyperparameter optimization, the GBR was updated to use a more robust loss function (huber) in place of the standard squared_error, a reduced learning rate of 0.05, and an increased number of boosting stages set to 150. The model now considers only the square root of the total number of features at each split, uses a subsample of 80% of the training data to introduce stochasticity, and enforces a higher minimum number of samples per leaf (set to 5) for better generalization. Additionally, the Huber loss smoothing parameter alpha was reduced slightly from 0.9 to 0.85 to make the model more sensitive to moderate outliers.

The KNN was reconfigured to use 29 neighbors for prediction, up from the default of 5, and to apply distance-based weighting, giving greater influence to closer neighbors. The search algorithm was explicitly set to kd_tree for faster neighbor lookup in low-dimensional spaces, with a reduced leaf size of 10 to allow for finer-grained tree structures. Additionally, the distance metric was changed from the general minkowski to the more specific l2 (Euclidean distance), improving consistency with the weighting strategy.

The RFR was enhanced by increasing the number of trees in the ensemble to 200 and enabling warm starts to allow incremental model building. The maximum depth of each tree was limited to 3 to reduce overfitting, and the number of features considered at each split was restricted using the square root strategy. Additionally, the criterion for splitting was changed to friedman_mse to better handle variance, the minimum number of samples required at a leaf node was raised to 7, and bootstrapping was disabled to ensure full dataset usage per tree.

To enhance performance, the SVR was adjusted with a lower regularization strength (C = 0.1) to reduce overfitting, and a significantly expanded cache size of 10,000 MB to accommodate more efficient computation. The kernel was changed from rbf to poly, enabling the model to capture more complex, nonlinear patterns. Additionally, the epsilon margin was tightened from 0.1 to 0.01 for more precise fits around target values, the coef0 parameter was increased slightly to 0.1 to influence the polynomial kernel’s flexibility, and the kernel coefficient gamma was switched from scale to auto. A looser convergence tolerance (tol = 0.1) was also introduced to speed up optimization.

The SGD was reconfigured to include elastic net regularization and a much smaller regularization strength (alpha) of 0.00001, allowing for more flexible fitting. Early stopping was enabled to reduce overfitting, and the total number of training iterations was reduced to 1,000 for computational efficiency. The initial learning rate (eta0) was lowered to 0.001 and coupled with an adaptive learning rate schedule, replacing the previous inverse scaling strategy. Additionally, the exponent used in learning rate scaling (power_t) was increased to 0.5, and the convergence tolerance (tol) was decreased to 0.0001 for finer convergence criteria.

The MLP was extensively refined to improve generalization and training stability. Early stopping was enabled to halt training when validation performance plateaued, and the maximum number of training iterations was substantially increased from 200 to 2,000 to allow more thorough convergence. The optimizer was switched from Adam to stochastic gradient descent (solver=’sgd’). The learning schedule was made adaptive, enabling the model to reduce the learning rate when progress slows, and the initial learning rate was lowered from 0.001 to 0.0001 for finer weight updates. Additionally, the batch size was explicitly set to 128 to better control gradient noise during training.

Lastly, the MLR model was adjusted to exclude the intercept term (fit_intercept = False), and constrain the model coefficients to be strictly positive (positive = True). Additionally, the convergence tolerance was relaxed from 1e-6 to 0.0001 to allow faster optimization without significantly compromising precision.

All default and tuned parameters are listed in appendix D. The results of each step of the hyperparameter tuning are listed in appendix E.

### Performance evaluation

The SGD reached the highest adjusted R^2^ (0.77), the highest ρ (0.83), the lowest MAPE (16.15%) and the lowest RMSE (44.13 mmHg), while it performed worse for the σ_ae_ (86.39) and the MAE (87.66 mmHg). Although most algorithms performed similarly, the SGD reached the lowest rank in four out of six parameters in our performance matrix from Table 1, and was hence selected as the best-performing algorithm for the given task.

**Table 1:** Performance matrix of tuned algorithms

		GBR	KNN	MLP	MLR	RFR	SGD	SVR
σ_**ae**_	BM	86.64	88.2	86.71	86.25	86.93	83.94	88.64
	TM	88.05	86.17	85.82	87.65	**79.88**	86.39	85.84
#		7	4	2	6	**1**	5	3
**MAPE in %**	BM	19.25	18.33	20.02	19.32	19.58	16.73	17.88
	TM	19.24	17.77	19.77	19.75	21.65	**16.15**	17.7
#		4	3	6	5	7	**1**	2
**Adjusted R**^**2**^	BM	0.7175	0.7197	0.7155	0.718	0.6962	0.7618	0.7426
	TM	0.7213	0.7396	0.7086	0.7093	0.6498	**0.7742**	0.7029
#		3	2	5	4	7	**1**	6
**RMSE in mmHg**	BM	49.45	49.17	49.58	49.41	51.26	45.32	47.16
	TM	49.11	47.4	50.18	50.16	55.04	**44.13**	50.68
#		3	2	5	4	7	**1**	6
**MAE in mmHg**	BM	84.63	85.2	86.5	85.03	85.63	85.22	86.76
	TM	85.55	82.18	85.28	86.21	**76.3**	87.66	82.04
#		5	3	4	6	**1**	7	2
**Spearman’s ρ**	BM	0.7528	0.7836	0.7653	0.7491	0.742	0.8224	0.7826
		[0.74; 0.7651]	[0.7722; 0.7946]	[0.753; 0.777]	[0.7361; 0.7616]	[0.7287; 0.7548]	[0.8127; 0.8315]	[0.7711; 0.7936]
	TM	0.7536	0.8014	0.7352	0.7345	0.7151	**0.8337**	0.7844
		[0.7408; 0.7658]	[0.7907; 0.8115]	[0.7216; 0.7483]	[0.7208; 0.7476]	[0.7006; 0.729]	**[0.8247; 0.8424]**	[0.773; 0.7953]
#		4	2	5	6	7	**1**	3
Sum of ranks		26	16	27	31	30	**16**	22

σ_ae_, adjusted R^2^, MAPE, RMSE, MAE, and Spearman’s ρ [95% CI] for each algorithm with default parameter values (Base Model (BM)) and tuned parameter values (Tuned Model (TM)) based on the test data set. The rank of the regressor for the considered performance metric is indicated in the column

**Fig. 4:**
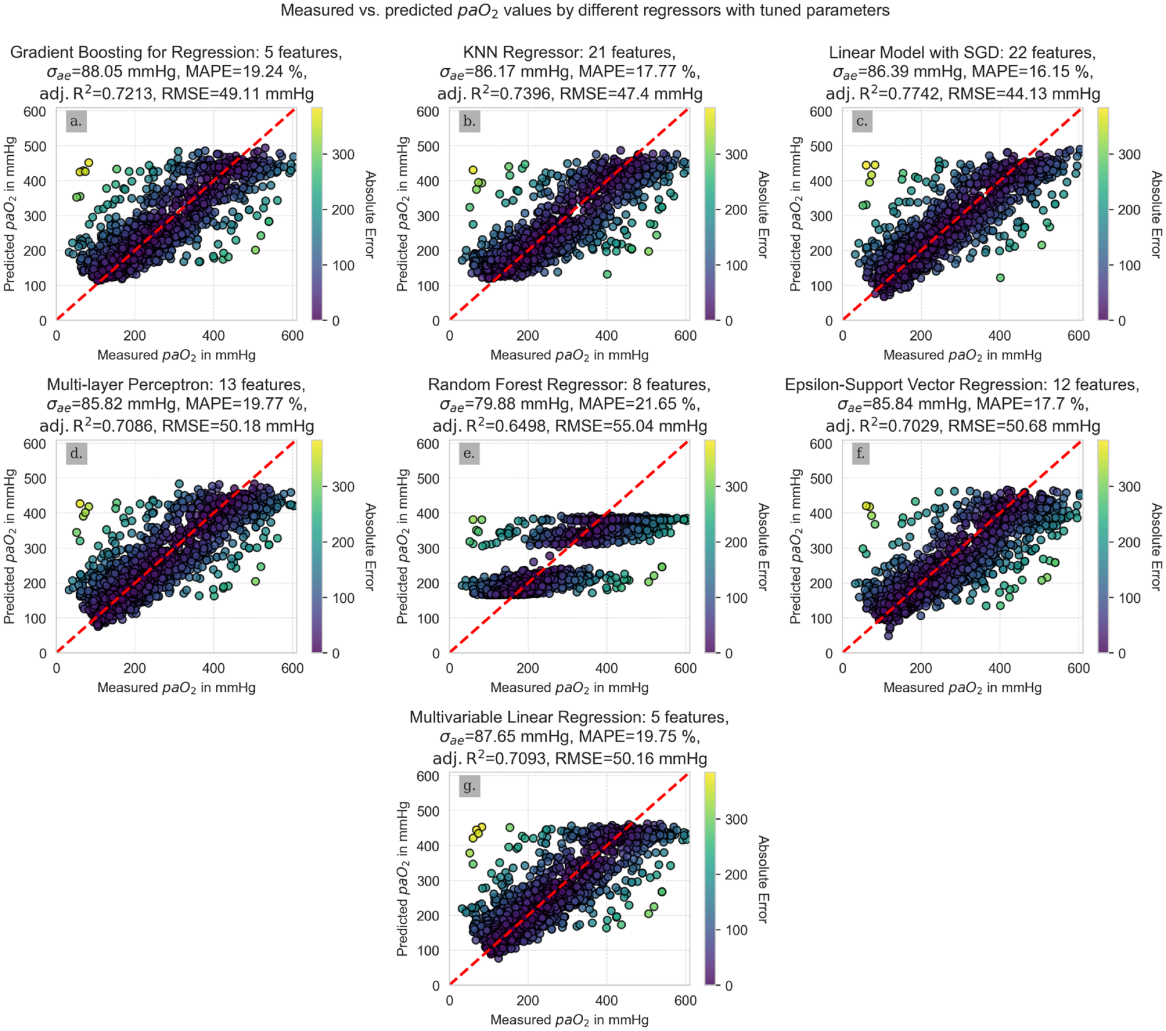
Scatterplots of measured vs. predicted paO_2_ values for different estimators. **a**. Gradient boosting for regression, **b**. Regression based on k-nearest neighbors, **c**. Linear model fitted by minimizing a regularized empirical loss with stochastic gradient descent, **d**. Multi-layer perceptron regressor, **e**. Random Forest regressor, **f**. Epsilon-support vector regression, **g**. Multivariable ordinary least squares linear regression

To visualize the correlation for each algorithm, the predicted and measured paO_2_ values of the test set were plotted against each other (Fig. 4).

### Further evaluation

**Fig. 5:**
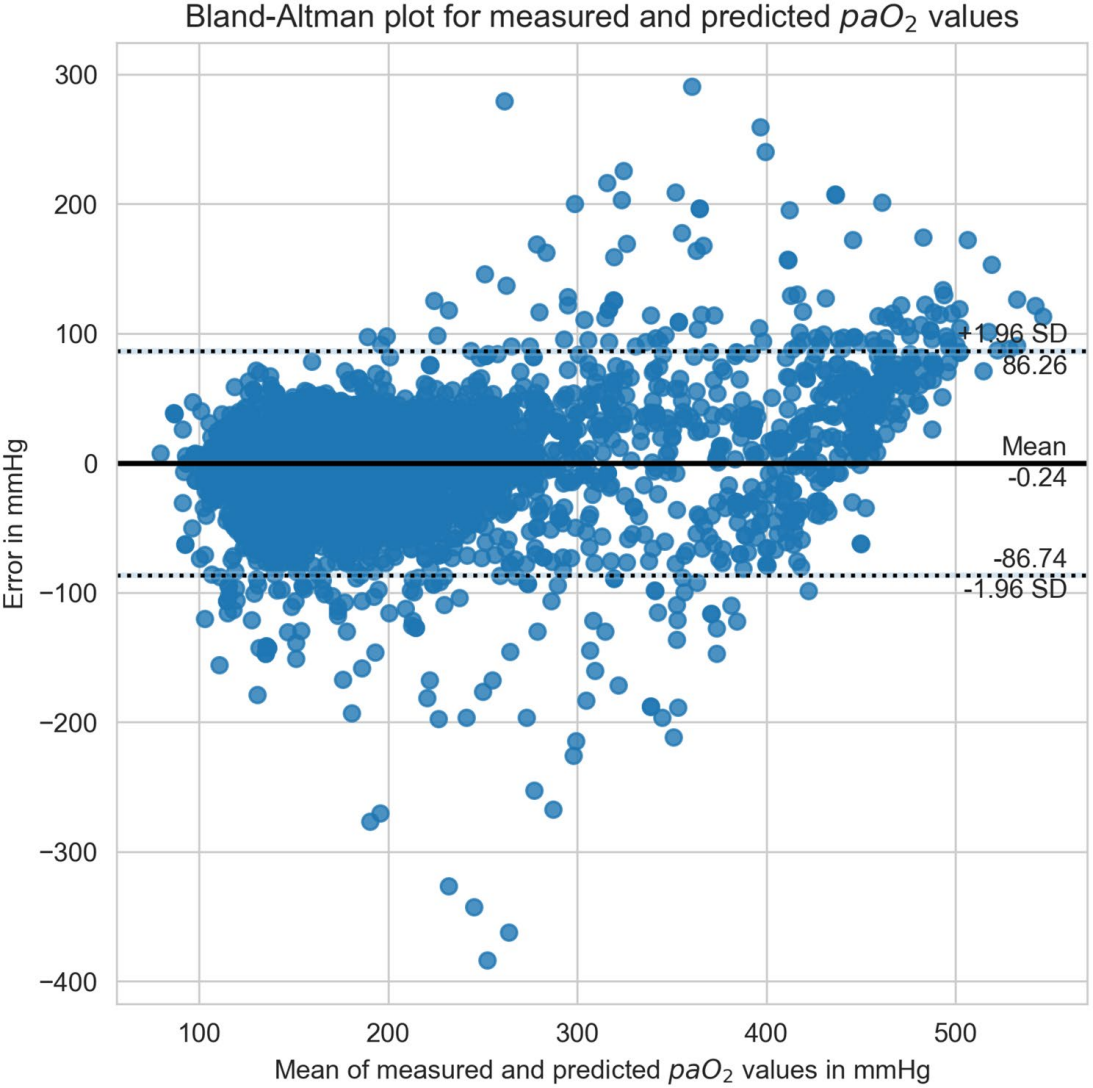
Bland-Altman plot for measured vs. predicted paO_2_ values. mean difference was −0.24 mmHg with limits of agreement (mean ±1.96 standard deviation) ranging from −86.74 mmHg to 86.26 mmHg

A Bland-Altman plot was generated to assess the agreement between the measured and predicted paO_2_ values (Fig. 5). The mean difference (bias) was minimal at −0.24 mmHg, indicating no substantial systematic error. The limits of agreement (mean ±1.96 standard deviation) ranged from −86.74 mmHg to 86.26 mmHg, reflecting the variability of prediction errors across the measurement range. While most errors clustered around zero, increased dispersion was observed at higher paO_2_ levels.

**Fig. 6:**
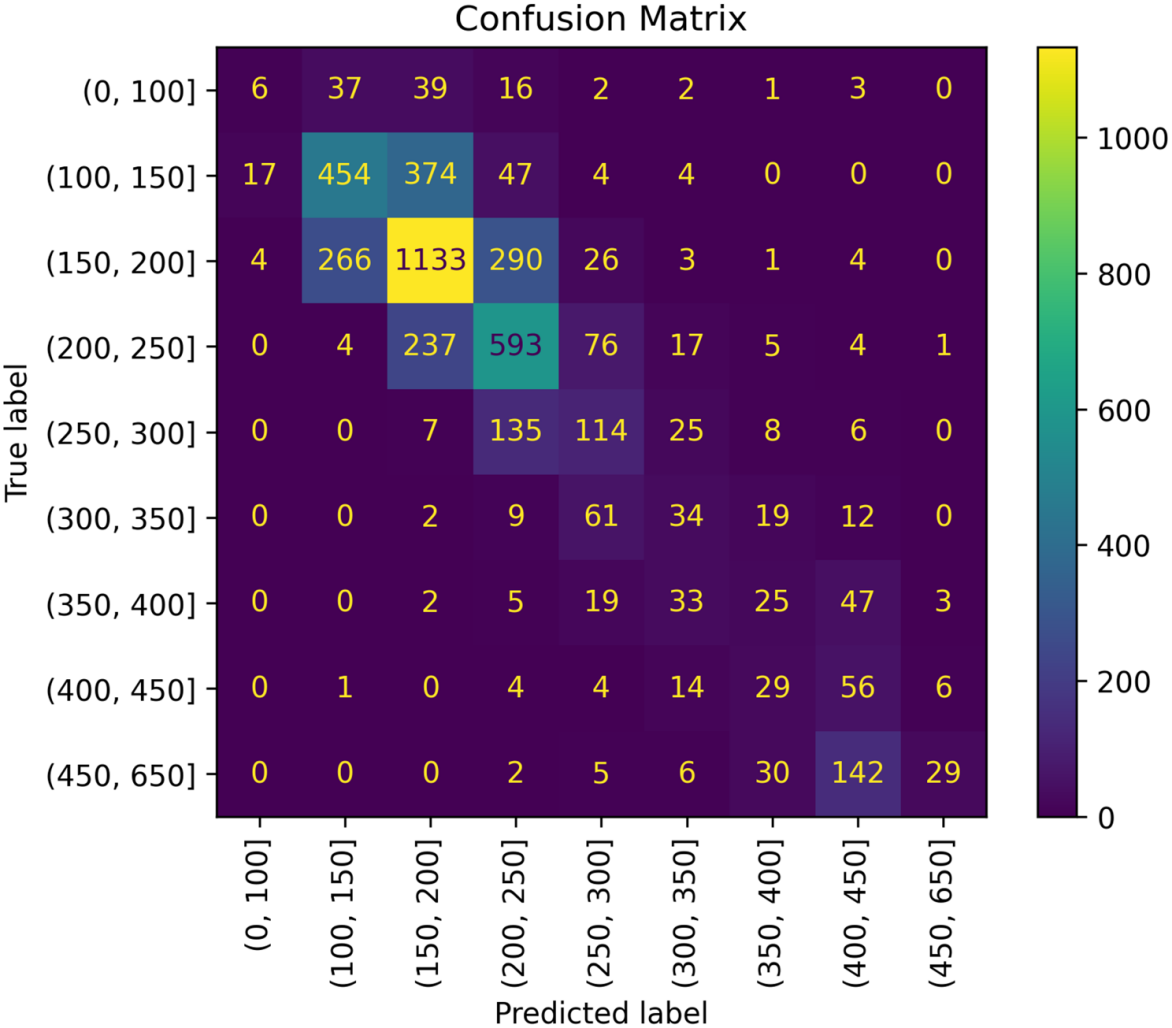
Confusion matrix for linear model fitted by minimizing a regularized empirical loss with stochastic gradient descent. Aggregated values per bin for observed and predicted values

In the next step, we constructed a confusion matrix using the test set, with bins defined in 50 mmHg intervals. Values below 100 mmHg and above 450 mmHg were grouped into single bins at each extreme (Fig. 6). Information about means and standard deviations for each bin is provided in table 2 (appendix J).

The tuned SGD overestimated paO_2_ values smaller than 100 mmHg and underestimated those larger than 450 mmHg.

**Fig. 7:**
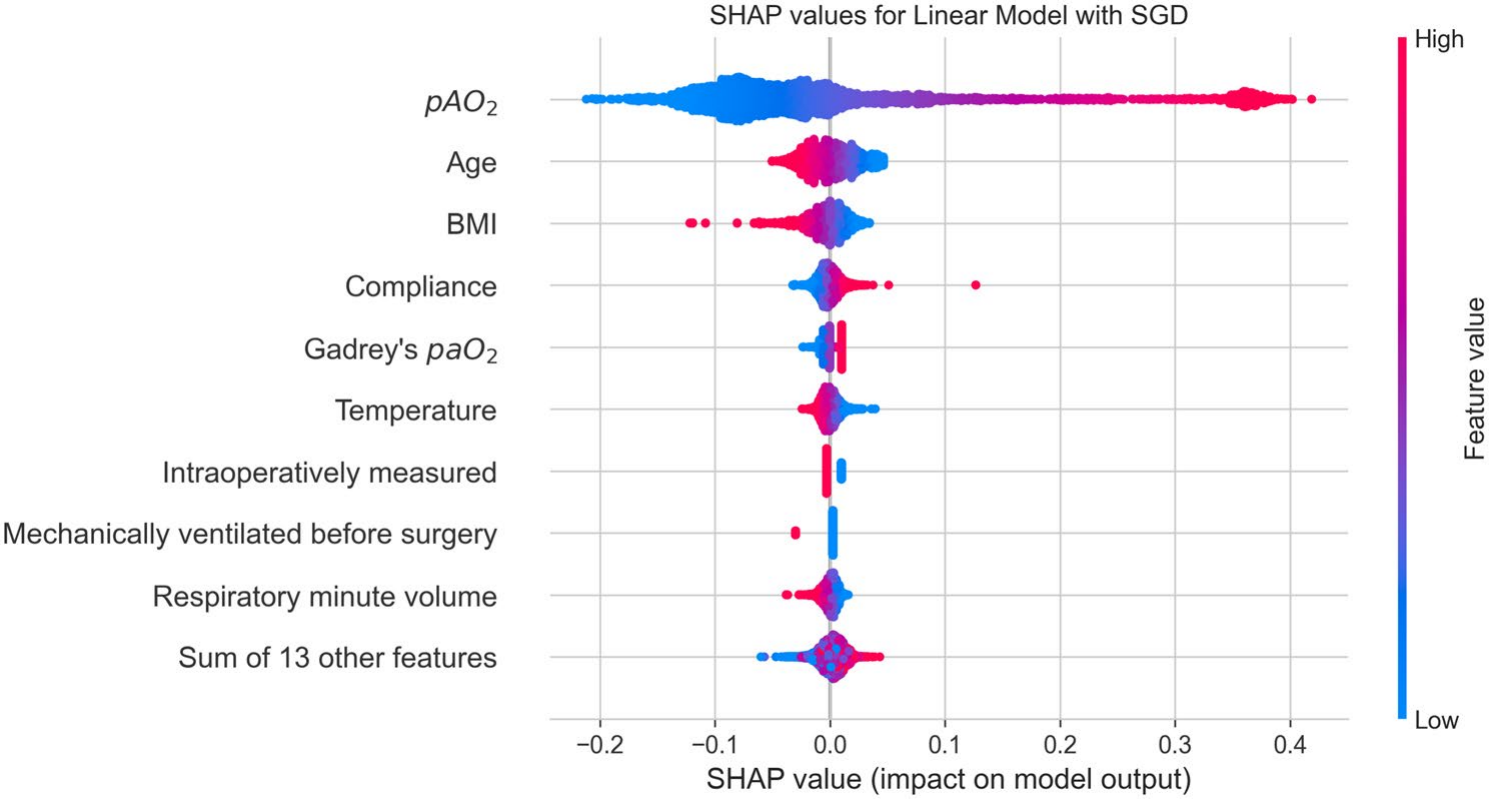
SHAP values. features are ordered by the mean absolute value of the SHAP values

We calculated SHAP values to see the contribution of each feature to the prediction, shown in Fig. 7. The feature with the highest SHAP value was pAO_2_, followed by age and BMI. They were followed by respiratory compliance, Gadrey’s paO_2_ values, temperature values, whether the ABG analysis was drawn intraoperatively, whether the patient was mechanically ventilated before surgery and the respiratory minute volume. The remaining 13 features were summed up, as their impact was considered low.

**Fig. 8:**
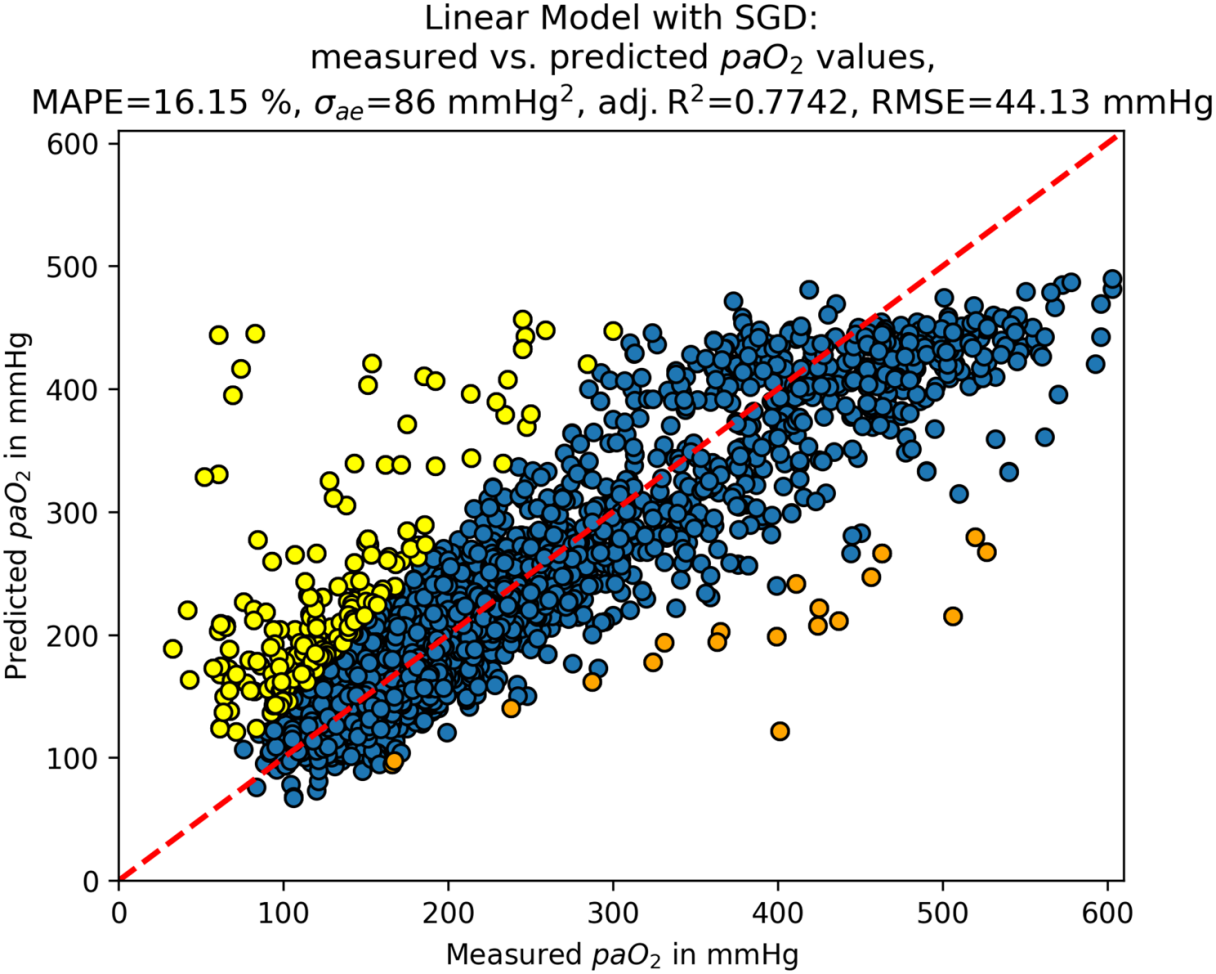
Evaluation of linear Model with stochastic gradient descent. Scatterplot of measured vs. predicted paO_2_ values with yellow being highly overestimated and orange being highly underestimated values

Percentage errors were calculated for all predicted paO_2_ values; the median was 1.09%. Q1 and Q3 were −9.99% and 10.69%, respectively, resulting in an IQR of 20.68%. We investigated all features for the predicted paO_2_ values that were identified as outliers based on their percentage errors (PE < −41.01%, PE > 41.71%). A total of 234 observations in 137 patients were highly overestimated, 20 observations in 19 patients were highly underestimated (Fig. 8). Among those, significant differences were found in two features.

Most of the underestimated values occurred between the second and third blood samples, but were closer to the second (mean index: 2.2). In contrast, overestimated values appeared later, near the time of the fourth blood sample (mean index: 3.8). A similar pattern was observed with respect to the timing of measurement: 77% of overestimated values occurred intraoperatively, whereas 55% of underestimated values were recorded during the perioperative period.

In the last step, we included the first measured p/F ratios in the best model. It improved further: In the test set, the adjusted R^2^ increased from 0.77 to 0.81, the MAPE decreased by almost two points to 14.5% and the RMSE decreased by four points to 40.3 mmHg. Additionally, the correlation coefficient was now 0.90 [0.90; 0.91], which was significantly better compared to all previously used proxies (FiO_2_, pAO_2_, and Gadrey’s paO_2_). Only the MAE increased by one point to 89.0 mmHg, while σ_ae_ remained almost the same (86.8 mmHg).

## Discussion

This study presents a machine learning algorithm for intraoperative live prediction of paO_2_ values in lung-healthy patients. Although the use of machine learning models in the medical context is not new at all, comparing different algorithms and applying them in a perioperative setting to predict arterial blood gas values is a novel and challenging approach, whose feasibility could be proven with satisfying results. From the selected algorithms, the tuned linear model fitted by minimizing a regularized empirical loss with stochastic gradient descent performed best. Its paO_2_ predictions were substantially better than the abilities of known proxies or calculation rules to extrapolate on paO_2_ values. These results allow a closer monitoring of administered oxygen without additional ABG analyses in lung-healthy patients.

To the best of our knowledge, this is the first study to present a machine learning algorithm for intraoperative live prediction of paO_2_ values in lung-healthy patients. The continuous assessment of paO_2_ is currently impossible. Frequent ABG analyses are time-consuming and potentially harmful for the patient due to the risk of infection and blood loss; in our data, they were collected every 1.7 hours (after applying exclusion criteria), providing many minutes for additional monitoring with live paO_2_ predictions. Therefore, FiO_2_ or paO_2_ have been used in the past to extrapolate to corresponding paO_2_ values [11–14, 18, 41]. In our dataset, the correlation between these values and the actual measured paO_2_ values was below 0.75, which was considerably worse than the tested algorithms. As paO_2_ does not directly correlate with the inspiratory fraction of oxygen but paO_2_ serves as one of the limited tools available to estimate a patient’s degree of hyperoxygenation, we aimed to develop a robust method for estimating perioperative paO_2_ values based on readily available input parameters. To construct this model, we collected routine data from intracranial neurosurgical operations. These surgeries are particularly well-suited for testing various algorithms for the prediction of intraoperative paO_2_ for several reasons: First, there is usually an isolated pathology in the neurocranium, and surgical procedures do not impact the thorax, ensuring that gas remains unaffected. Second, arterial catheters are part of the standard monitoring for patients undergoing craniotomy at our clinic. Thirdly, craniotomies typically span three to five hours of surgical time, during which multiple ABG analyses are drawn, providing a substantial and rich dataset for analysis.

Gadrey et al. were the first to introduce a new equation of paO_2_ calculation, based on two constant values and the SpO_2_ value [30]. With SpO_2_ being the only measured variable, the outcome has a natural maximum of 132.8 mmHg. Thus, the equation is not suitable to model hyperoxemia. Although others used it for predicting arterial partial pressure of oxygen which might only be applicable for a physiological range [42, 43]. Additionally, the correlation coefficient was relatively low at 0.26 and smaller than the correlation coefficient of FiO_2_ or pAO_2_ to the measured paO_2_ value.

We used machine learning algorithms to model the relatively complex interactions between perioperative and sociodemographic values. The model using a stochastic gradient descent performed best. Two of its main advantages are its computational efficiency and its many options for hyperparameter tuning to fit a specific problem. One of its drawbacks is the sensitivity to feature scaling, requiring all input features to be scaled equally.

This study faces some limitations. First, our findings are not generalizable to patients with relevant pulmonary dysfunction, such as chronic obstructive pulmonary disease, asthma, lung cancer, or acute respiratory distress syndrome. This cohort was specifically selected to exclude these comorbidities, as our first aim was to investigate which machine learning models would be generally suitable for this task and how well they would perform predictively. Second, besides the surgical procedure and the main diagnosis, we did not consider other comorbidities when training and fitting the different algorithms. The reason for this was the need to develop a generalizable algorithm, which could be used in a broad range of patients with different kinds of comorbidities but the same type of surgical procedure. Still, with MAPEs of 16% and 14% (when including the first measured p/F ratio), our algorithm yielded very accurate results. Third, we only included patients with invasive ventilation and general anesthesia who received at least two ABG analyses. Fourth, the study size might be too small for deep learning methods to deploy their full potential [44, 45]. Fifth, external validation was not possible at the current stage, as no independent dataset was available. We are currently preparing a separate dataset to facilitate validation in future work. Sixth, in the range of hypoxic and normoxic paO_2_ values (up to 100 mmHg), we rather overestimate the paO_2_ value, whereas in severe hyperoxia (more than 450 mmHg) we rather underestimate the true value (Fig. 8). In the lower paO_2_ range, there were insufficient data available to effectively train the algorithm, limiting the reliability of predictions in this segment. However, this also reflects the clinical reality that hypoxemic values were rarely observed in this stable cohort with predominantly healthy lung function, indicating that hypoxemia was not a relevant issue in this population. Moreover, hypoxemia can typically be detected in clinical practice through non-invasive pulse oximetry (e.g., fingertip sensors), making precise paO_2_ estimation in this range less critical. It is also important to consider the clinical relevance of prediction errors: for instance, an error of 40 mmHg may be of little consequence when paO_2_ is 400 mmHg but could be clinically significant when paO_2_ is closer to 100 mmHg. However, such borderline or hypoxemic values occurred infrequently in this dataset, further limiting their impact on clinical decision-making in this specific cohort. Seventh, the algorithm predicts some serious outliers. They may arise due to various factors, such as technical variability, mislabeling of blood samples, or inaccurate measurements (e.g., temperature sensors placed outside the bladder). Since these data are collected during routine clinical care, they are inherently subject to error. Therefore, our algorithm should be regarded as a conceptual approach for the continuous estimation of paO_2_, which still requires validation against ABG measurements.

## Conclusion

In this study, we demonstrate that machine learning algorithms can be utilized to predict paO_2_ values for a range between 100 to 450 mmHg. Our SGD did not only achieve the highest adjusted R^2^ of 0.77 and 0.80 (when including the first measured p/F ratio), the lowest MAPE of 16%, but also the highest correlation coefficient and the smallest RMSE. Although some papers exist that extrapolate paO_2_ values, this is the first model for live prediction of paO_2_ values with satisfactory results. Such a tool might support medical staff to continuously estimate paO_2_ values to enhance monitoring and prevention of hyperoxemia in a perioperative setting. Continuous in-silico prediction of paO_2_ levels might also enhance estimation of the total excess of oxygen during patient treatment, allowing researchers to better investigate its effects.

Our next study will use these results for a quasi-real time prediction in the same patient collective to evaluate the effect of excessive oxygen on postoperative complications. Besides that, future studies are needed to validate our method in other patient collectives and clinical scenarios.

## Electronic supplementary material

Below is the link to the electronic supplementary material.

Supplementary Material 1

Supplementary Material 2

Supplementary Material 3

Supplementary Material 4

Supplementary Material 5

Supplementary Material 6

Supplementary Material 7

Supplementary Material 8

Supplementary Material 9

Supplementary Material 10

## Data Availability

The code and rendered notebook files supporting the conclusion of this article are publicly available in the git repository: https://github.com/abeckerp/pao2-prediction.

## Abbreviations

σ_ae_: Standard Deviation of Absolute Errors
ABG: Arterial Blood Gas
ASA: American Society of Anesthesiologists
BM: Base Model
BMI: Body Mass Index
CV: Cross-Validation
FiO_2_: Fraction of Inspired Oxygen
GBR: Gradient Boosting for Regression
IQR: Interquartile Range
KNN: Regression based on k-nearest neighbors
MAE: Mean Absolute Error
MAPE: Mean Absolute Percentage Error
MLP: Multi-layer Perceptron Regressor
MLR: Multivariable ordinary least squares Linear Regression
N: Number of Surgeries
n: Number of Observations (multiple per surgery)
p/F: paO_2_/FiO_2_
pAO_2_: Alveolar Oxygen Partial Pressure
paO_2_: Partial Arterial Pressure of Oxygen
PE: Percentage Error
Q1 / Q3: First / Third Quartile
RFE: Recursive Feature Elimination
RFR: Random Forest Regressor
RMSE: Root Mean Squared Error
SGD: Linear model fitted by minimizing a regularized empirical loss with stochastic gradient descent
SHAP: SHapley Additive exPlanations
SpO_2_: Peripheral Oxygen Saturation
SVR: Epsilon-Support Vector Regression
TM: Tuned Model
