# Supplementary material for: Comparing supervised machine learning algorithms for the prediction of partial arterial pressure of oxygen during craniotomy
Source: BMC Med Inform Decis Mak. 2025 Sep 3;25:326. doi: 10.1186/s12911-025-03148-8 (PMC12406590; doi:10.1186/s12911-025-03148-8)
Supplement: Supplementary file 1 — Supplementary Material 1 [file 12911_2025_3148_MOESM1_ESM.pdf]

Appendix A: Formulas used for Features

- Alveolar gas equation: $pAO_2 = \frac{FiO_2}{100} * (713.622 - 47) - \frac{CO_2}{0.82}$, assuming a prevailing atmospheric pressure of 713.622 mmHg and a saturated vapor pressure of water of 47 mmHg [1]. End-tidal carbon dioxide values were used instead of arterial partial pressure of carbon dioxide.
- Static compliance: $C_{stat} = \frac{RMV}{RR} * \frac{1000}{P_{plat}-PEEP} = \frac{V}{P_{plat}-PEEP},\ where\ V = \frac{RMV}{RR} * 1000$ with RMV as the respiratory minute volume, RR as the respiratory rate, P_{plat} as the plateau pressure in cm $H_2 0$ and $PEEP$ as the positive end-expiratory pressure in cm $H_2 0$ [2]
- $Gadrey'spaO_2 = \frac{23400}{\frac{1}{SpO_2}-0.99}^{\frac{1}{3}}$ [3]
- Feature and label scaling based on training data using the min-max normalization: $x_{scaled} = s * (max - min) + min,\ with\ s = (x - x_{min})/(x_{max} - x_{min})\ and\ min = 0,\ max = 1$ [4, 5]

References

[1] Sharma, S., Hashmi, M. F. & Burns, B. *Alveolar Gas Equation*. StatPearls (StatPearls Publishing, Treasure Island (FL), 2019). URL https://www.ncbi.nlm.nih.gov/pubmed/29489223.

[2] Desai, J. P. & Moustarah, F. *Pulmonary Compliance*. StatPearls (StatPearls Publishing, Treasure Island (FL), 2019). URL https://www.ncbi.nlm.nih.gov/pubmed/30855908.

[3] Gadrey, S. M. *et al.* Imputation of partial pressures of arterial oxygen using oximetry and its impact on sepsis diagnosis. *Physiological measurement* **40**, 115008 (2019). URL https://www.ncbi.nlm.nih.gov/pubmed/31652430.

[4] Pedregosa, F. *et al.* Scikit-learn: Machine learning in python. *Journal of machine learning research: JMLR* **12**, 2825–2830 (2011). URL http://jmlr.org/papers/v12/pedregosa11a.html.

[5] Han, J., Kamber, M. & Pei, J. Data mining concepts and techniques third edition. *University of Illinois at Urbana-Champaign Micheline Kamber Jian Pei Simon Fraser University* (2012). URL https://www.academia.edu/download/43034828/Data_Mining_Concepts_And_Techniques_3rd_Edition.pdf.
