# Supplementary material for: Comparing supervised machine learning algorithms for the prediction of partial arterial pressure of oxygen during craniotomy
Source: BMC Med Inform Decis Mak. 2025 Sep 3;25:326. doi: 10.1186/s12911-025-03148-8 (PMC12406590; doi:10.1186/s12911-025-03148-8)
Supplement: Supplementary file 2 — Supplementary Material 2 [file 12911_2025_3148_MOESM2_ESM.pdf]

Appendix B: List of Features

Full list of features:

- identifier: identifier for surgery,
- idx: incrementing number of the measurement (discrete value),
- fio2: inspiratory fraction of oxygen (continuous value),
- co2: end-tidal carbon dioxide in mmHg (continuous value),
- spo2: peripheral capillary oxygen saturation in % (continuous value),
- rmv: respiratory minute volume in liter (continuous value),
- respiratory_rate: respiratory rate in 1/minute (discrete value),
- compliance: ventilation compliance in millilitermillibar (continuous value),
- paO2_measured: measured paO_2 value in mmHg (continuous value),
- first_horowitz: first paO_2/FiO_2 ratio in mmHg (continuous value),
- last_horowitz: last measured paO_2/FiO_2 ratio in mmHg (continuous value),
- pAO2: pAO_2 calculated value by the alveolar gas equation in mmHg (continuous value),
- systolic: systolic value in mmHg (discrete value),
- diastolic: diastolic value in mmHg (discrete value),
- mean_art_press: mean arterial pressure in mmHg (discrete value),
- heart_rate: heart rate in 1/minute (discrete value),
- temperature: temperature value in degree celsius (continuous value),
- ph: last measured pH-value (different sampling sites) (continuous value),
- hemoglobin: last measured hemoglobin value in gdL (different sampling sites) (continuous value),
- case_number: a patient's case number during a hospital stay
- already_intubated: indicates whether a patient was already intubated before surgery (dichotomous value),

- not_extubated: indicates whether a patient was not extubated after surgery (dichotomous value),
- bmi: a patient's body mass index (continuous value),
- age: age in years (continuous value),
- los: postoperative length-of-stay in days (continuous value),
- creatinine: pre-operative creatinine value in milligramdeciliter (continuous value),
- time_to_incision: time from intubation to incision in minutes (continuous value),
- time_to_end: time from suture to extubation in minutes (continuous value),
- mv_time: invasive mechanical ventilation time in minutes (continuous value),
- incision_closure_time: time in minutes from incision to closure (continuous value),
- gadrey: paO_2 values calculated by Gadrey et al. in mmHg [30] (continuous value),
- sex_male: indicates a male patient (dichotomous value),
- asa: patient's ASA score (discrete value),
- timepoint_intraop: indicates whether the measurement was done intraoperatively (dichotomous value)

Features not eligible for recursive feature elimination, hyperparameter tuning and evaluation:

- paO2_measured,
- los
- identifier,
- incision_closure_time,
- mv_time,
- time_to_incision,
- time_to_end,
- not_extubated,
- first_horowitz,
- last_horowitz,

- case_number
