# Supplementary material for: Comparing supervised machine learning algorithms for the prediction of partial arterial pressure of oxygen during craniotomy
Source: BMC Med Inform Decis Mak. 2025 Sep 3;25:326. doi: 10.1186/s12911-025-03148-8 (PMC12406590; doi:10.1186/s12911-025-03148-8)
Supplement: Supplementary file 3 — Supplementary Material 3 [file 12911_2025_3148_MOESM3_ESM.pdf]

Appendix C: Inclusion and Exclusion Criteria

Observations with negative paO_2 values were excluded. Then, surgeries with invalid parameters were excluded; those were missing or negative data on postoperative length-of-stay, missing or measured creatinine values of <0.2 mg/dL, a BMI <14 or >60 kg/m^2, an initial intraoperative p/F ratio <300 mmHg (indicating impaired lung oxygenation), and surgeries with less than 5 min incision-to-closure time or mechanical ventilation times and negative times to incision or to end. Secondly, observations with negative ABG values or missing FiO_2, CO_2, or SpO_2 were excluded. Additional exclusion criteria consisted of measured hemoglobin levels <5 g/dL [1, 2], recorded blood pH values <6.8, a heart rate ¡20/min, a respiratory rate <5/min, a respiratory minute volume <2 l/min. We also removed observations with body temperature measurements <32°C or >44°C, which we interpreted as a disconnected temperature sensor. Patients, which were older than 100 years were excluded as well. Additionally, observations with measured paO_2 values <60 mmHg were excluded if hemoglobin values exceeded 7 g/dL and SpO_2 levels exceeded 95 %, as these values were likely attributed to venous blood gas analysis rather than arterial (Figure 1).

References

[1] Hemoglobin level. *Nursing in critical care* **15**, 34 (2020).

[2] Lundsgaard-Hansen, P., Doran, J. E. & Blauhut, B. Is there a generally valid, minimum acceptable hemoglobin level? *Infusionstherapie* **16**, 167–175 (1989).

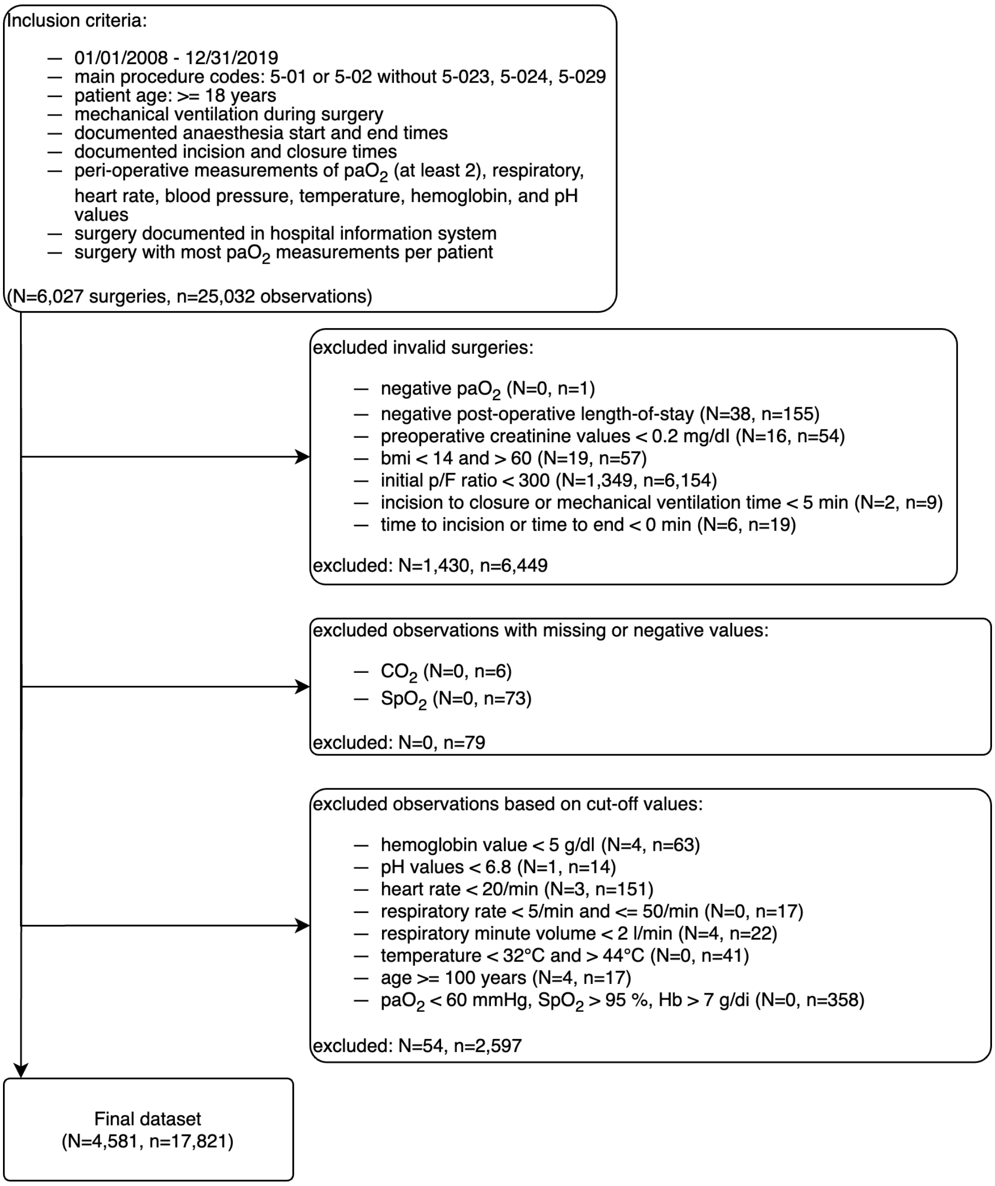

Fig. 1 Patient flowchart with inclusion and exclusion criteria.
