# Supplementary material for: Comparing supervised machine learning algorithms for the prediction of partial arterial pressure of oxygen during craniotomy
Source: BMC Med Inform Decis Mak. 2025 Sep 3;25:326. doi: 10.1186/s12911-025-03148-8 (PMC12406590; doi:10.1186/s12911-025-03148-8)
Supplement: Supplementary file 4 — Supplementary Material 4 [file 12911_2025_3148_MOESM4_ESM.pdf]

Appendix D: Default and Final Parameters of Estimators

Table 1 Gradient Boosting for Regression

	Default Parameters	Hyperparameters1	Hyperparameters2	Best Parameters
alpha	0.9		[0.85, 0.9]	0.85
ccp_alpha	0.0			0.0
criterion	friedman_mse			friedman_mse
init				
learning_rate	0.1	[0.05, 0.1]	[0.05]	0.05
loss	squared_error	['squared_error']	['huber']	huber
max_depth	3	[3, 4]	[3]	3
max_features		['sqrt', None]	['sqrt']	sqrt
max_leaf_nodes				
min_impurity_decrease	0.0			0.0
min_samples_leaf	1	[3, 5, 10]	[5]	5
min_samples_split	2			2
min_weight_fraction_leaf	0.0			0.0
n_estimators	100	[150, 200, 250]	[150, 200]	150
n_iter_no_change				
random_state	42			42
subsample	1.0	[0.8, 1.0]	[0.8]	0.8
tol	0.0001			0.0001
validation_fraction	0.1			0.1
verbose	0			0
warm_start	False			False

Default, hyper- and best parameters.

Table 2 K-nearest Neighbors Regression

	Default Parameters	Hyperparameters	Best Parameters
algorithm	auto	['ball_tree', 'kd_tree', 'brute', 'auto']	kd_tree
leaf_size	30	[5 10 15 20 25 30 35 40 45 50 55]	10
metric	minkowski	['minkowski', 'l1', 'l2']	l2
metric_params			
n_jobs			
n_neighbors	5	[5 13 21 29 37 45 53]	29
p	2	[1, 2]	2
weights	uniform	['uniform', 'distance']	distance

Default, hyper- and best parameters.

Table 3 Random Forest Regressor

	Default Parameters	Hyperparameters	Best Parameters
bootstrap	True	[True, False]	False
ccp_alpha	0.0		0.0
criterion	squared_error	['squared_error', 'friedman_mse']	friedman_mse
max_depth		[3, 9, 16, 23, 30]	3
max_features	1.0	['sqrt', 'log2', 0.6, 1.0]	sqrt
max_leaf_nodes			
max_samples			
min_impurity_decrease	0.0		0.0
min_samples_leaf	1	[1 4 7]	7
min_samples_split	2	[2, 4, 6]	2
min_weight_fraction_leaf	0.0		0.0
monotonic_cst			
n_estimators	100	[50, 87, 125, 162, 200]	200
n_jobs			
oob_score	False		False
random_state	42		42
verbose	0		0
warm_start	False	[True, False]	True

Default, hyper- and best parameters.

Table 4 Epsilon-Support Vector Regression

	Default Parameters	Hyperparameters1	Hyperparameters2	Best Parameters
C	1.0	[0.01, 0.1, 1.0, 10, 100]	[0.01, 0.1, 1.0, 10, 100]	0.1
cache_size	200	[10000]	[10000]	10000
coef0	0.0	[0.0, 0.1, 0.2]	[0.0, 0.1, 0.2]	0.1
degree	3		[3, 4]	3
epsilon	0.1	[0.001, 0.01, 0.1, 1.0, 10]	[0.001, 0.01, 0.1, 1.0, 10]	0.01
gamma	scale	['auto', 'scale']	['auto', 'scale']	auto
kernel	rbf	['rbf', 'sigmoid']	['poly']	poly
max_iter	-1			-1
shrinking	True			True
tol	0.001	[0.0001, 0.001, 0.01, 0.1, 1.0]	[0.0001, 0.001, 0.01, 0.1, 1.0]	0.1
verbose	False			False

Default, hyper- and best parameters.

Table 5 Linear model with SGD

	Default Parameters	Hyperparameters	Best Parameters
alpha	0.0001	[1e-05, 0.0001, 0.001]	1e-05
average	False		False
early_stopping	False	[True]	True
epsilon	0.1	[0.01, 0.1]	0.1
eta0	0.01	[0.001, 0.01]	0.001
fit_intercept	True		True
l1_ratio	0.15	[0.15, 0.3, 0.5]	0.15
learning_rate	invscaling	['invscaling', 'adaptive']	adaptive
loss	squared_error	['squared_error']	squared_error
max_iter	100000000	[1000]	1000
n_iter_no_change	5		5
penalty	l2	['elasticnet']	elasticnet
power_t	0.25	[0.25, 0.5]	0.5
random_state	42		42
shuffle	True		True
tol	0.001	[0.0001, 0.001]	0.0001
validation_fraction	0.1	[0.1]	0.1
verbose	0		0
warm_start	False		False

Default, hyper- and best parameters.

Table 6 Multivariable ordinary least squares Linear Regression

	Default Parameters	Hyperparameters	Best Parameters
copy_X	True	[False, True]	True
fit_intercept	True	[False, True]	False
n_jobs			
positive	False	[False, True]	True
tol	1e-06	[1e-07, 1e-06, 1e-05, 0.0001]	0.0001

Default, hyper- and best parameters.

Table 7 Multi-layer Perceptron Regressor

	Default Parameters	Hyperparameters1	Hyperparameters2	Hyperparameters3	Best Parameters
activation	relu	['relu', 'tanh', 'logistic']	['relu', 'tanh', 'logistic']	['relu', 'tanh', 'logistic']	relu
alpha	0.0001	[0.0001, 0.001]	[0.0001, 0.001]	[0.0001, 0.001]	0.0001
batch_size	auto	['auto', 64, 128]	['auto', 64, 128]	['auto', 64, 128]	128
beta_1	0.9				0.9
beta_2	0.999				0.999
early_stopping	False	[True]	[True]	[True]	True
epsilon	1e-08				1e-08
hidden_layer_sizes	(100,)	[(100,), (256, 128), (64,), (256, 128, 64), (64, 32)]	[(100,), (256, 128), (64,), (256, 128, 64), (64, 32)]	[(100,), (256, 128), (64,), (256, 128, 64), (64, 32)]	(100,)
learning_rate	constant	['constant', 'invscal-ing', 'adaptive']			adaptive
learning_rate_init	0.001	[0.001, 0.0001]	[0.001, 0.0001]	[0.001, 0.0001]	0.0001
loss	squared_error				squared_error
max_fun	15000				15000
max_iter	200	[2000]	[200]	[200]	2000
momentum	0.9	[0.5, 0.7, 0.9]			0.9
n_iter_no_change	10				10
nesterovs_momentum	True				True
power_t	0.5	[0.3, 0.5, 0.7]			0.5
random_state	42				42
shuffle	True				True
solver	adam	['sgd']	['adam']	['lbfgs']	sgd
tol	0.0001	[0.0001, 0.001]	[0.0001, 0.001]	[0.0001, 0.001]	0.0001
validation_fraction	0.1				0.1
verbose	False				False
warm_start	False				False

Default, hyper- and best parameters.
