# Supplementary material for: Comparing supervised machine learning algorithms for the prediction of partial arterial pressure of oxygen during craniotomy
Source: BMC Med Inform Decis Mak. 2025 Sep 3;25:326. doi: 10.1186/s12911-025-03148-8 (PMC12406590; doi:10.1186/s12911-025-03148-8)
Supplement: Supplementary file 6 — Supplementary Material 6 [file 12911_2025_3148_MOESM6_ESM.pdf]

Appendix F: Different systems for data extraction and analysis

Table 1 Different systems for data extraction and analysis

	System 1	System 2	System 3
**Description**	Ubuntu 22.04.02 LTS, Linux 5.15.0	macOS 15.5 with arm64	Ubuntu 24.04.2 LTS, Linux 6.8.0 with 160 CPUs per task and 150 GB memory
**Python version**	3.10.12	3.12.11	3.12.3
**Django version**	4.1.7		
**Requests version**	2.28.2		
**Pandas version**	1.5.3	2.3.0	2.3.0
**Matplotlib version**		3.10.3	
**Seaborn version**		0.13.2	
**Sklearn version**		1.7.0	1.7.0
**Numpy version**		2.3.1	2.3.1
**Scipy version**		1.11.3	1.11.4
**Statsmodels version**		0.14.4	
**Joblib version**			1.5.1
**Yaml version**		6.0.2	
**Quarto version**	1.3.361	1.7.32	
**Jupyter notebooks version**	6.5.3	7.4.3	
**Shap version**		0.48.0	
**Pingouin version**		0.5.5	

The following libraries were used with a random state and seed of 42 wherever applicable:

- Data extraction: django, requests
- Data preparation: pandas
- Data visualization: matplotlib, seaborn, shap, pingouin
- Feature selection: pandas, numpy, sklearn
- Hyperparameter tuning: sklearn, numpy
- Feature Importance: shap
- Statistical tests: numpy, scipy, statsmodels
- Authoring system: Quarto with Jupyter notebooks
