# Supplementary material for: Comparing supervised machine learning algorithms for the prediction of partial arterial pressure of oxygen during craniotomy
Source: BMC Med Inform Decis Mak. 2025 Sep 3;25:326. doi: 10.1186/s12911-025-03148-8 (PMC12406590; doi:10.1186/s12911-025-03148-8)
Supplement: Supplementary file 9 — Supplementary Material 9 [file 12911_2025_3148_MOESM9_ESM.pdf]

Appendix I: Descriptive Statistics

[1] indicates one-time measurements per patient.
[2] indicates multiple measurements per patient (feature).
[1] indicates one-time measurements per patient.
[2] indicates multiple measurements per patient (feature).
[1] indicates one-time measurements per patient.
[2] indicates multiple measurements per patient (feature).

Table 1 Descriptive Statistics

	count	mean	std	min	25%	50%	75%	max
[1]**age in years**	4,581	54.25	16.1	18.0	43.0	55.0	67.0	95.0
[1]**already intubated before anaesthesia**	4,581	0.08	0.28	0.0	0.0	0.0	0.0	1.0
[1]**ASA class**	4,581	2.67	0.75	1.0	2.0	3.0	3.0	5.0
[1]**BMI in kg/m^2**	4,581	25.07	4.38	14.81	22.23	24.51	27.34	58.44
[1]**pre-operative creatinine in mg/dL**	4,581	0.95	0.34	0.2	0.8	0.9	1.0	9.3
[1]**initially measured p/F ratio**	4,581	461.53	99.77	300.0	390.69	458.75	517.29	1,534.46
[1]**incision to closure time in min**	4,581	242.61	103.32	12.0	174.0	232.0	298.0	1,194.25
[1]**postoperative (in-hospital) length of stay in days**	4,581	11.62	11.07	1.0	6.0	7.0	12.0	175.0
[1]**mechanical ventilation time in min**	4,581	353.21	119.73	58.98	273.0	344.75	420.5	1,382.0
[1]**no extubation after surgery**	4,581	0.23	0.42	0.0	0.0	0.0	0.0	1.0
[1]**sex (0=female)**	4,581	0.44	0.5	0.0	0.0	0.0	1.0	1.0
[2]**CO_2 in mmHg**	17,821	34.64	3.35	0.66	33.0	35.0	37.0	57.0
[2]**static pulmonary compliance in ml/cm H_2O**	17,821	48.15	11.46	4.99	40.4	47.22	55.0	217.19
[2]**diastolic blood pressure in mmHg**	17,821	59.65	10.61	13.0	53.0	59.0	65.0	251.0
[2]**FiO_2**	17,821	0.48	0.17	0.2	0.37	0.43	0.5	1.0
[2]**Gadrey's paO_2 in mmHg**	17,821	106.34	18.6	28.18	91.64	105.2	132.76	132.76
[2]**heart rate in 1/min**	17,821	58.97	13.61	29.0	50.0	57.0	65.0	180.0
[2]**hemoglobin in g/dL**	17,821	12.07	1.81	5.3	11.0	12.3	13.3	18.0
[2]**mean arterial pressure in mmHg**	17,821	79.55	11.06	14.0	73.0	78.0	85.0	255.0
[2]**pAO_2 in mmHg**	17,821	278.33	114.75	95.25	206.54	244.54	292.54	648.41
[2]**measured paO_2 in mmHg**	17,821	211.64	91.36	32.7	155.0	187.1	232.7	626.1
[2]**pH**	17,821	7.41	0.05	6.99	7.38	7.42	7.45	7.98
[2]**respiratory rate in 1/min**	17,821	11.11	2.28	5.0	10.0	11.0	12.0	39.0
[2]**respiratory minute volume in L**	17,821	5.63	1.37	2.0	4.7	5.48	6.4	16.8
[2]**SpO_2 in %**	17,821	98.7	1.25	49.13	98.0	99.0	100.0	100.0
[2]**systolic blood pressure in mmHg**	17,821	115.09	15.26	17.0	106.0	114.0	123.0	283.0
[2]**temperature in °C**	17,821	36.3	0.77	32.0	35.88	36.37	36.8	38.99
[2]**measurement taken intraoperatively**	17,821	0.73	0.45	0.0	0.0	1.0	1.0	1.0

Table 2 Descriptive Statistics - test data

	count	mean	std	min	25 %	50 %	75 %	max
[1]**age in years**	1,145	53.99	16.03	18.0	42.0	55.0	67.0	95.0
[1]**already intubated before anaesthesia**	1,145	0.09	0.28	0.0	0.0	0.0	0.0	1.0
[1]**ASA class**	1,145	2.67	0.75	1.0	2.0	3.0	3.0	5.0
[1]**BMI in kg/m^2**	1,145	25.12	4.51	15.24	22.22	24.61	27.4	58.44
[1]**pre-operative creatinine in mg/dL**	1,145	0.95	0.4	0.29	0.8	0.9	1.0	7.8
[1]**initially measured p/F ratio**	1,145	460.01	99.82	301.19	389.16	454.84	517.59	1,092.24
[1]**incision to closure time in min**	1,145	248.07	101.11	13.0	181.25	238.0	302.0	702.25
[1]**postoperative (in-hospital) length of stay in days**	1,145	11.89	11.23	1.0	6.0	7.0	13.0	90.0
[1]**mechanical ventilation time in min**	1,145	358.59	117.14	58.98	277.0	349.75	427.0	814.75
[1]**no extubation after surgery**	1,145	0.24	0.43	0.0	0.0	0.0	0.0	1.0
[1]**sex (0=female)**	1,145	0.42	0.49	0.0	0.0	0.0	1.0	1.0
[2]**CO_2 in mmHg**	4,564	34.73	3.34	4.0	33.0	35.0	37.0	50.68
[2]**static pulmonary compliance in ml/cm H_2O**	4,564	47.85	11.56	5.86	40.56	46.99	54.54	217.19
[2]**diastolic blood pressure in mmHg**	4,564	59.53	10.18	14.0	53.0	59.0	65.0	215.0
[2]**FiO_2**	4,564	0.48	0.17	0.2	0.38	0.43	0.5	1.0
[2]**Gadrey's paO_2 in mmHg**	4,564	105.93	18.62	44.73	91.64	105.2	132.76	132.76
[2]**heart rate in 1/min**	4,564	58.63	12.92	29.0	50.0	57.0	65.0	140.0
[2]**hemoglobin in g/dL**	4,564	12.07	1.77	5.5	11.0	12.3	13.2	17.8
[2]**mean arterial pressure in mmHg**	4,564	79.42	10.85	14.0	72.0	78.0	85.0	215.0
[2]**pAO_2 in mmHg**	4,564	279.53	115.15	95.25	208.19	246.97	293.13	648.41
[2]**measured paO_2 in mmHg**	4,564	211.92	93.1	32.7	154.0	188.0	232.58	602.9
[2]**pH**	4,564	7.41	0.05	6.99	7.38	7.42	7.44	7.63
[2]**respiratory rate in 1/min**	4,564	11.07	2.25	5.0	10.0	11.0	12.0	35.0
[2]**respiratory minute volume in L**	4,564	5.58	1.33	2.0	4.64	5.4	6.4	14.3
[2]**SpO_2 in %**	4,564	98.67	1.24	79.9	98.0	99.0	100.0	100.0
[2]**systolic blood pressure in mmHg**	4,564	114.98	15.07	17.0	106.0	114.0	123.0	216.0
[2]**temperature in °C**	4,564	36.33	0.76	32.06	35.9	36.4	36.84	38.99
[2]**measurement taken intraoperatively**	4,564	0.73	0.44	0.0	0.0	1.0	1.0	1.0

Table 3 Descriptive Statistics - training data

	count	mean	std	min	25 %	50 %	75 %	max
[1]**age in years**	3,436	54.34	16.13	18.0	43.0	55.0	67.0	94.0
[1]**already intubated before anaesthesia**	3,436	0.08	0.28	0.0	0.0	0.0	0.0	1.0
[1]**ASA class**	3,436	2.67	0.76	1.0	2.0	3.0	3.0	5.0
[1]**BMI in kg/m^2**	3,436	25.05	4.33	14.81	22.3	24.49	27.18	54.86
[1]**pre-operative creatinine in mg/dL**	3,436	0.95	0.32	0.2	0.8	0.9	1.0	9.3
[1]**initially measured p/F ratio**	3,436	462.04	99.77	300.0	392.03	459.81	516.92	1534.46
[1]**incision to closure time in min**	3,436	240.79	104.0	12.0	173.0	230.25	295.75	1194.25
[1]**postoperative (in-hospital) length of stay in days**	3,436	11.53	11.01	1.0	6.0	7.0	12.0	175.0
[1]**mechanical ventilation time in min**	3,436	351.42	120.55	76.75	271.25	343.0	418.82	1382.0
[1]**no extubation after surgery**	3,436	0.23	0.42	0.0	0.0	0.0	0.0	1.0
[1]**sex (0=female)**	3,436	0.45	0.5	0.0	0.0	0.0	1.0	1.0
[2]**CO_2 in mmHg**	13,257	34.62	3.36	0.66	33.0	35.0	36.99	57.0
[2]**static pulmonary compliance in ml/cm H_2O**	13,257	48.25	11.43	4.99	40.33	47.27	55.18	132.92
[2]**diastolic blood pressure in mmHg**	13,257	59.69	10.75	13.0	53.0	59.0	65.0	251.0
[2]**FiO_2**	13,257	0.48	0.17	0.2	0.37	0.43	0.5	1.0
[2]**Gadrey's paO_2 in mmHg**	13,257	106.48	18.59	28.18	91.64	105.2	132.76	132.76
[2]**heart rate in 1/min**	13,257	59.08	13.83	30.0	50.0	57.0	65.0	180.0
[2]**hemoglobin in g/dL**	13,257	12.06	1.82	5.3	11.0	12.3	13.3	18.0
[2]**mean arterial pressure in mmHg**	13,257	79.6	11.13	36.0	73.0	78.0	85.0	255.0
[2]**pAO_2 in mmHg**	13,257	277.91	114.61	97.88	206.12	243.96	292.5	642.0
[2]**measured paO_2 in mmHg**	13,257	211.54	90.76	41.3	155.0	187.0	232.7	626.1
[2]**pH**	13,257	7.41	0.05	7.05	7.38	7.42	7.45	7.98
[2]**respiratory rate in 1/min**	13,257	11.12	2.29	5.0	10.0	11.0	12.0	39.0
[2]**respiratory minute volume in L**	13,257	5.65	1.39	2.01	4.7	5.5	6.41	16.8
[2]**SpO_2 in %**	13,257	98.71	1.26	49.13	98.0	99.0	100.0	100.0
[2]**systolic blood pressure in mmHg**	13,257	115.13	15.33	47.0	106.0	114.0	124.0	283.0
[2]**temperature in °C**	13,257	36.29	0.77	32.0	35.87	36.36	36.8	38.8
[2]**measurement taken intraoperatively**	13,257	0.73	0.45	0.0	0.0	1.0	1.0	1.0
