# Supplementary material for: Comparing supervised machine learning algorithms for the prediction of partial arterial pressure of oxygen during craniotomy
Source: BMC Med Inform Decis Mak. 2025 Sep 3;25:326. doi: 10.1186/s12911-025-03148-8 (PMC12406590; doi:10.1186/s12911-025-03148-8)
Supplement: Supplementary file 10 — Supplementary Material 10 [file 12911_2025_3148_MOESM10_ESM.pdf]

Appendix J: Mean and Standard Deviations of Binned Values

Table 1 Mean and Standard Deviations of Binned Values

	N observed	Mean paO_2 value and standard deviation (observed values)	Mean paO_2 value and standard deviation (predicted values)
**(0, 100]**	106	82.82 ± 15.95	169.61 ± 67.54
**(100, 150]**	900	131.93 ± 13.3	151.19 ± 31.84
**(150, 200]**	1727	175.72 ± 14.44	178.57 ± 31.34
**(200, 250]**	937	222.06 ± 13.89	218.1 ± 34.31
**(250, 300]**	295	269.25 ± 13.86	260.42 ± 42.85
**(300, 350]**	137	322.17 ± 15.37	310.58 ± 54.83
**(350, 400]**	134	375.18 ± 14.04	360.99 ± 64.15
**(400, 450]**	114	421.38 ± 13.96	387.56 ± 58.23
**(450, 650]**	214	496.59 ± 34.01	418.55 ± 41.43
